# Epidemiology of lower urinary tract symptoms in a cross-sectional, population-based study: The status in China

**DOI:** 10.1097/MD.0000000000011554

**Published:** 2018-08-24

**Authors:** Jian-Ye Wang, Limin Liao, Ming Liu, Budiwan Sumarsono, Min Cong

**Affiliations:** a Department of Urology, Beijing Hospital; b Department of Urology, China Rehabilitation Research Center, Capital Medical University, Beijing, China; c Astellas Pharma Singapore Pte. Ltd., Singapore; d Astellas Pharma China, Inc., Beijing, China.

**Keywords:** China, epidemiology, lower urinary tract symptoms, overactive bladder, prevalence

## Abstract

Lower urinary tract symptoms (LUTS) are reported to affect over half of all adults, and they are associated with significantly impaired quality of life (QOL). We performed a population-based study to evaluate the overall prevalence and impact of LUTS including overactive bladder (OAB) in adults aged ≥40 years in China.

Adults aged ≥40 years were eligible to participate in this internet-based survey, provided that they had the ability to access the internet, to use a computer and to read the local language. The survey contained questions relating to International Continence Society (ICS) symptom definitions, the International Prostate Symptom Score (IPSS) and the Overactive Bladder Symptom Score (OABSS). The primary study objective was to determine the prevalence of LUTS using the ICS 2002 symptom definition.

Among the 4136 respondents, 2080 (50.3%) were men and 1347 (32.6%) were aged ≥60 years. LUTS prevalence according to ICS criteria was 60.3% in men and 57.7% in women. All 3 ICS symptom groups (voiding, storage, and postmicturition) were present in 22.8% of women and 24.2% of men, making this the most common combination of ICS symptom groups. The most bothersome symptoms were terminal dribble and nocturia. According to IPSS scores, 32.9% of participants had at least moderate symptoms. The prevalence of OAB was 23.9%. The presence of LUTS—particularly all 3 ICS symptom groups—was associated with reduced sexual QOL in women, reduced satisfaction with erectile function in men, higher anxiety and depression scores, and reduced health-related QOL (physical health and mental health domains). The overall percentage of participants with LUTS visiting healthcare professionals for urinary symptoms was 38%.

In conclusion, LUTS affect the majority of adults aged ≥40 years in China, and prevalence increases with increasing age. LUTS are associated with impaired QOL and mental health, but fewer than half of individuals in China with LUTS seek healthcare for their symptoms. There is therefore a need to improve awareness and treatment of the condition.

ClinicalTrials.gov identifier: NCT02618421

## 1 Introduction

The term lower urinary tract symptoms (LUTS) encompasses a wide variety of different symptoms. According to the International Continence Society (ICS), they can be classified into 3 categories: voiding, storage, and postmicturition.^[1]^ Although LUTS are not usually life-threatening, under-recognition, which contributes to suboptimal treatment, and under-treatment are significant issues as they increase the overall symptom burden. Epidemiological studies are valuable in providing an insight into real-world prevalence and impact of LUTS. The use of ICS terminology when conducting such studies can be helpful in ensuring that the results of different epidemiological studies are comparable.

International studies have shown that LUTS affect over half of all adults, with increasing prevalence among older age groups.^[2,3]^ In China, a population-based survey conducted across 5 regions, using the International Consultation on Incontinence Questionnaire Male/Female Lower Urinary Tract Symptoms Long Form, reported that 61% of men and women aged ≥18 years had at least 1 LUTS.^[4]^

Individuals with LUTS have significantly impaired quality of life (QOL).^[5–8]^ For example, in a study of Chinese LUTS patients, poorer health-related quality of life (HRQOL) was evident in relation to the general health domain, vitality domain and physical component summary, compared with normal values for the Hong Kong population.^[5]^ A greater impact on QOL was evident in patients with nocturia, frequency, urgency, or mixed urinary incontinence, and in those affected at a young age. LUTS can have significant pharmacoeconomic impact, with costs arising through the purchase of incontinence pads and reduced workplace productivity.^[9–11]^ In addition, individuals with LUTS have increased risk of mental health problems such as depression and anxiety.^[10,12]^ Consequently, considerable benefits can be gained from optimal therapy of LUTS. However, many individuals with symptoms do not consult a healthcare professional for treatment.^[13]^

We performed a population-based study to evaluate the overall prevalence and impact of LUTS including overactive bladder (OAB) in adults aged ≥40 years in China, Taiwan, and South Korea. The combined results from all 3 countries are available elsewhere.^[14–16]^ The prevalence data indicate that LUTS affects approximately 60% of the combined population, with a slightly higher rate in men than women (62.8% vs 59.6%; *P = *.004).^[14]^ LUTS prevalence was shown to increase significantly with age (*P = *.001). OAB was present in 19.5% of men and 22.1% of women and, as with LUTS, there was a significant increase in OAB prevalence with increasing age (*P = *.001).^[15]^ HRQOL was also impaired among individuals with LUTS, with a corresponding increase in levels of anxiety and depression.^[16]^ Here, by presenting data from this study relating to 1 country, we aim to provide a more detailed insight into LUTS prevalence and burden specifically in China.

## 2 Methods

This international study was conducted using an internet-based survey between June 2 and July 20, 2015. The methods have been published in full previously.^[14]^ Therefore, in this publication we are providing a short description only.

Adults aged ≥40 years living in the general community were eligible to participate, provided that they had the ability to access the internet, to use a computer (with help, if required) and to read the local language. Individuals who had a urinary tract infection within the preceding month were excluded as were women who were pregnant at the time of the survey. Participants were selected via consumer survey panels, with email invitations sent to prevalidated individuals. To address possible bias, the demographic profile of the study population (age, sex, and socioeconomic factors) was actively managed within each survey panel.

The survey contained questions relating to ICS symptom definitions,^[1]^ the International Prostate Symptom Score (IPSS) and the Overactive Bladder Symptom Score (OABSS). In addition, HRQOL, the Hospital Anxiety and Depression Scale (HADS), the International Index of Erectile Function (IIEF, completed by men only), and Sexual Quality of Life—Female (SQOL-F, completed by women only) were included.

The primary study objective was to determine the prevalence of LUTS using the ICS 2002 symptom definition. Secondary objectives included assessment of the associations of LUTS with HRQOL, HADS scores, and SQOL-F and IIEF scores. The degree of symptom-specific bother of LUTS was assessed using a Likert scale: not at all ([score 0], a little bit [score 1], somewhat [score 2], quite a bit [score 3] or a great deal [score 4]). A score <2 suggested that there was no or only a little bit of associated bother while a score ≥2 indicated “somewhat or greater bother” and a score ≥3 was the most severe degree of bother, “quite a bit or greater.” In addition, the prevalence of OAB was measured using OABSS.

For assessment of the percentage of the population with a particular LUTS characteristic and with a 95% confidence interval, it was calculated that ≥384 participants would be required per age group, increasing to 1920 for 5 different age groups. Approximately, 28% of data were expected to be nonevaluable (e.g., missing/inconsistent information) and, consequently, total enrollment of at least 2667 respondents was planned. Initial data analyses were based on descriptive statistics. There was no imputation for missing values: respondents who completed the relevant sections of the questionnaire were included in the analysis and the rest were excluded. As it was a web survey with logical checks, it was confirmed that all answers were coherent. Post-hoc statistical comparisons were chosen for consistency with the EpiLUTS study,^[2]^ with comparisons performed principally using the Chi-square test. Differences were considered to be statistically significant with *P*-values ≤.05. A step-wise multiple logistic regression analysis was performed with treatment seeking as the binary independent variable. A total of 29 independent variables were included in the model, including continuous, categorical and binary variables. The final set of variables was chosen according to clinical relevance. Data analyses were performed using R (3.2.1) (R Foundation for Statistical Computing, Vienna, Austria), SPSS Base 15 (SPSS Inc., Chicago, IL) and Microsoft Excel 2016 (Microsoft Corp., Redmond, WA).

Institutional review board approval was not considered necessary for this study as it was based on a survey. However, principles of the Declaration of Helsinki were followed, and the study was performed in compliance with Good Clinical Practice and market research guidelines. Prior to participation in the study, all participants provided informed consent.

## 3 Results

A total of 399,215 email invitations were sent to Chinese individuals. Informed consent was obtained from 13,819 individuals, of whom 9683 were excluded either because the survey quota had already been filled or because the individual had completed the survey rapidly/superficially (i.e., unreliable results). Thus, surveys from 4136 respondents were analyzed; 2080 respondents (50.3%) were men and 1347 (32.6%) were aged ≥60 years (Table 1).

**Table 1:**
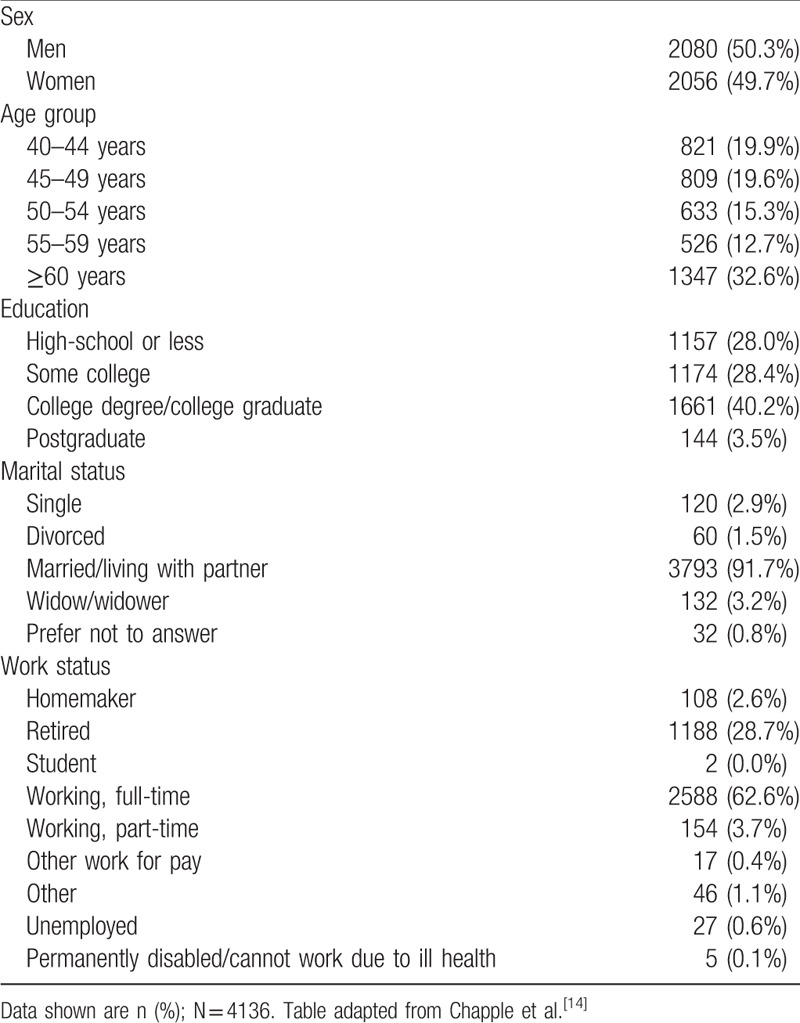
Demographic characteristics of Chinese participants.

**Figure 1:**
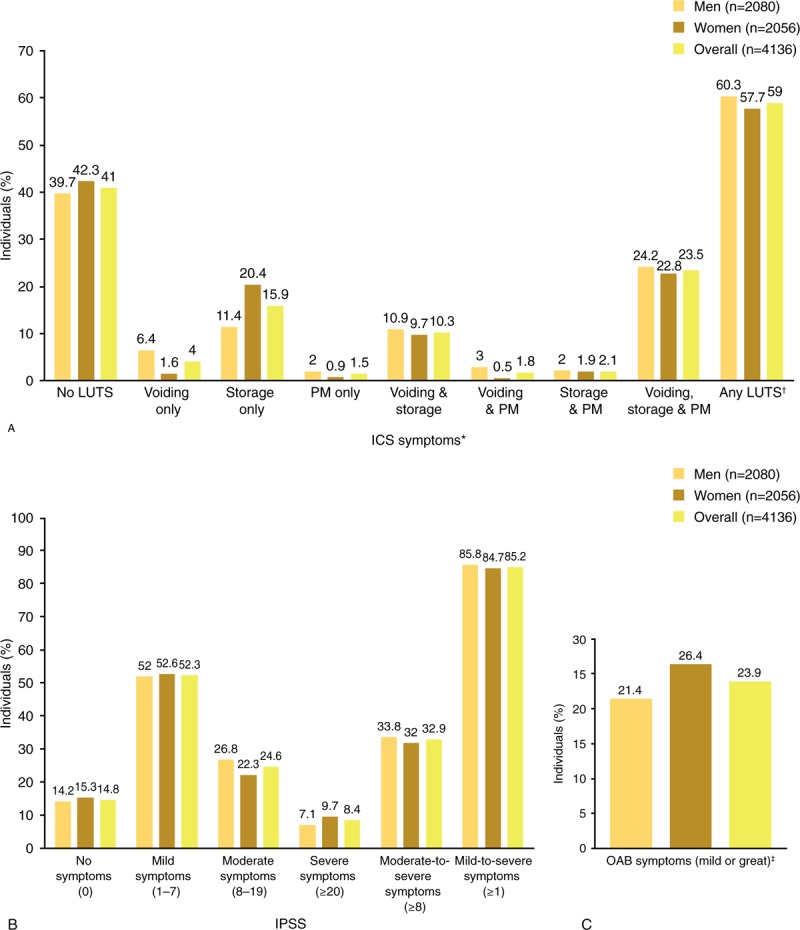
Prevalence of LUTS in China according to ICS 2002 symptom definition (A) and IPSS definition (B), and prevalence of OAB according to OABSS (C). ^∗^Prevalence based on symptom frequency of rating ≥2 and nocturia ≥2 episodes. ^†^Prevalence based on ≤1 of voiding, storage, or postmicturition (PM) symptoms. ^‡^The overall OABS score is the sum of the scores for 4-individual items, and the diagnostic criteria for OAB are a total OABSS of ≥3 with an urgency score (for Question 3) of 2 or more. ICS = International Continence Society, IPSS = International Prostate Symptom Score, LUTS = lower urinary tract symptoms, OAB = overactive bladder, OABSS = Overactive Bladder Symptom Score, PM = postmicturition.

**Figure 2:**
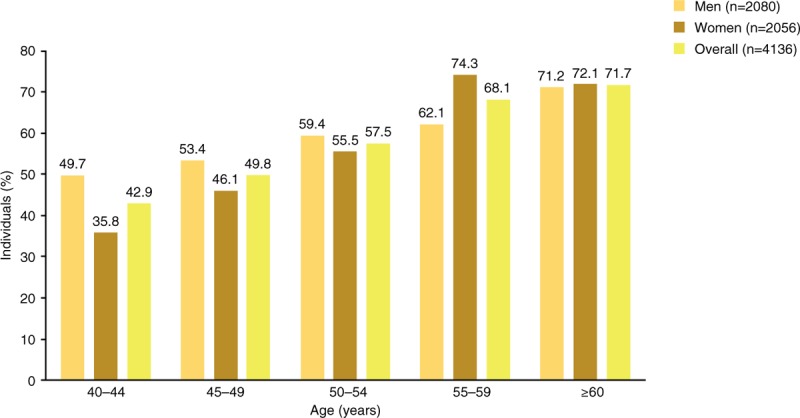
LUTS prevalence in China by age. LUTS = lower urinary tract symptoms.

LUTS prevalence according to ICS criteria was slightly higher in men (60.3%) than in women (57.7%; Fig. 1A). All 3 ICS symptom groups (voiding, storage, and postmicturition) were present in 23.5% of individuals (women: 22.8%, men: 24.2%), making this the most common combination of ICS symptom groups (Fig. 1A). The second most common symptom type was storage symptoms only (15.9%); this was more prevalent in women than in men (20.4% vs 11.4%). A combination of storage and voiding symptoms was third most common, with a prevalence of 10.3% (women: 9.7%, men: 10.9%). Voiding symptoms only were more prevalent in men than women (6.4% vs 1.6%). In both men and women, LUTS prevalence showed a tendency to increase with age (*P* < .001, Chi-square test) (Fig. 2).

**Figure 3:**
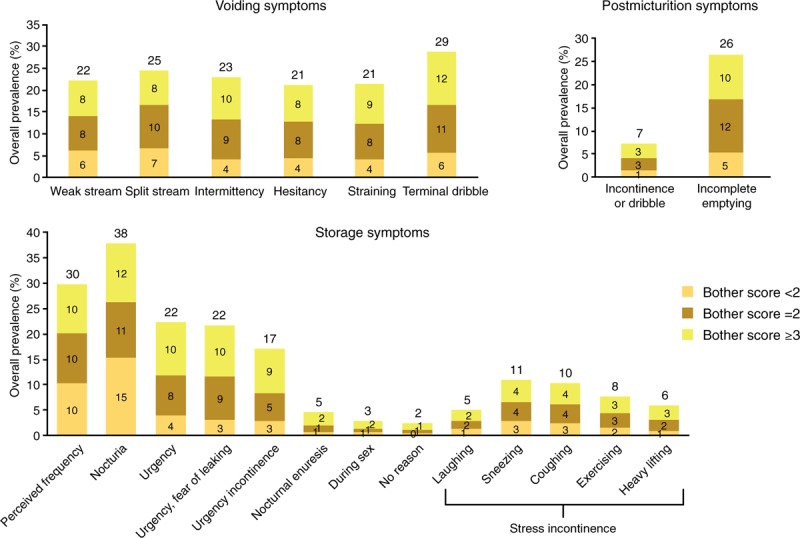
Prevalence in China of specific symptoms and bother: men and women. Bother score based on percentage of patients who experienced the symptom; numbers at top of bar represent overall prevalence. Score <2: no or a little bit of bother; score ≥2: somewhat or greater bother; score ≥3: quite a bit or greater bother.

Symptoms with the highest overall prevalence were nocturia (38%), perceived frequency (30%), terminal dribble (29%), and incomplete emptying (26%) (Fig. 3). In men, the 2 most prevalent symptoms were nocturia (35%) and terminal dribble (32%); those in women were nocturia (41%) and perceived frequency (31%). The most bothersome symptoms (those with the largest numbers of participants experiencing “quite a bit or greater” bother) were terminal dribble and nocturia (Fig. 3). These were followed by intermittency, incomplete emptying, perceived frequency, urgency, and urgency with fear of leaking. Therefore, 4 of the 7 most bothersome symptoms were related to storage. The overall prevalence of stress urinary incontinence (SUI) was 22.0%, with rates of 16.8% in men and 27.2% in women.

According to IPSS scores, 85.2% of participants had at least mild symptoms. Prevalence of moderate or greater symptoms (IPSS score ≥8) was 32.9% (Fig. 1B). For men and women with moderate symptoms, IPSS QOL was “unhappy” or worse in 29% of participants, and the corresponding percentage among individuals with severe symptoms was 58%. Sex-specific IPSS QOL data are shown in Table 2; only minor differences were evident between men and women.

**Table 2:**
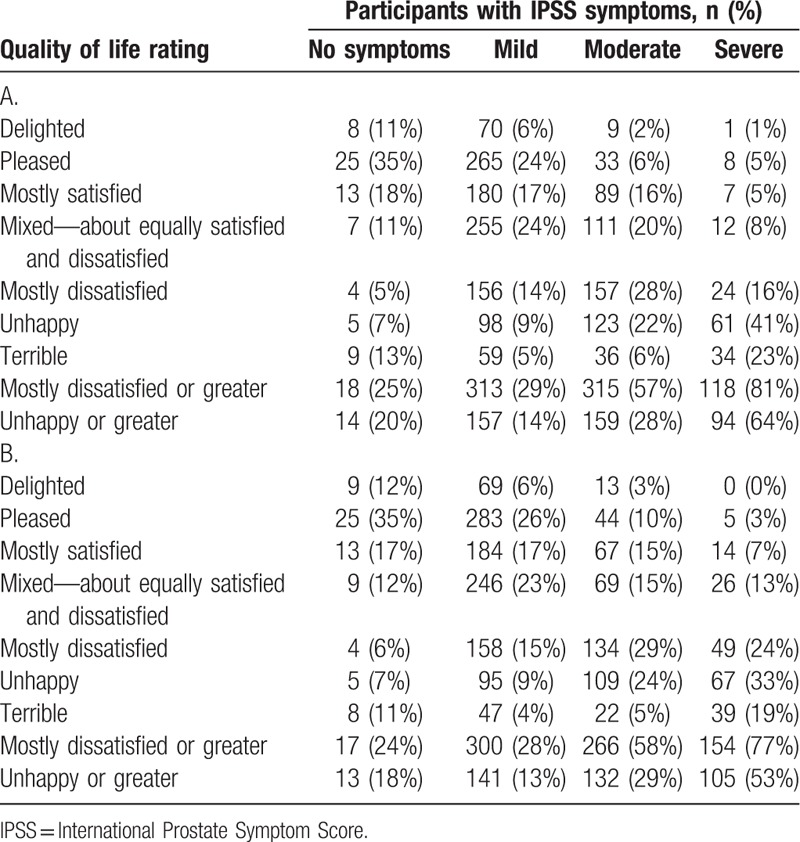
International Prostate Symptom Score quality of life according to International Prostate Symptom Score symptom severity in Chinese men (A; n = 1856) and women (B; n = 1814).

The prevalence of OAB, as determined by OABSS, was 23.9%: 21.4% in men and 26.4% in women (Fig. 1). In comparison, the overall prevalence of storage symptoms was 51.8%. All comorbid conditions investigated were associated with significantly higher OAB prevalence rates (*P* < .001; Table 3). The presence of diabetes was associated with the greatest increase in OAB prevalence, from 18.6% in individuals without the condition to 50.7% in those with diabetes. The impact of comorbid conditions on OAB prevalence was similar in men and women (data not shown).

**Table 3:**
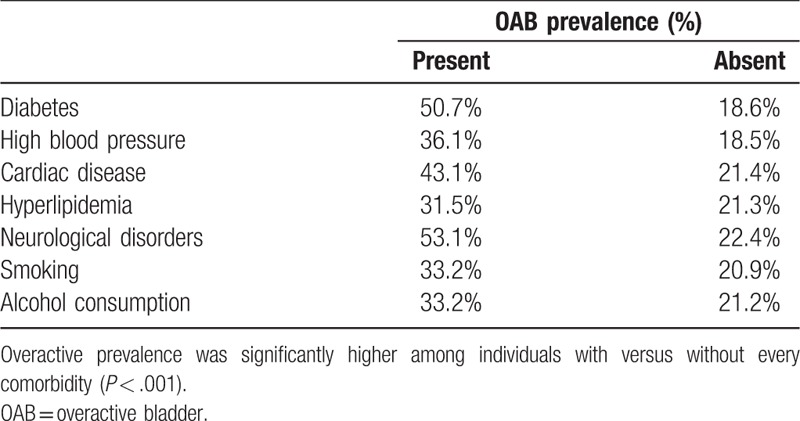
Overactive bladder prevalence in China by comorbidities (data for men and women combined, n = 4136).

**Figure 4:**
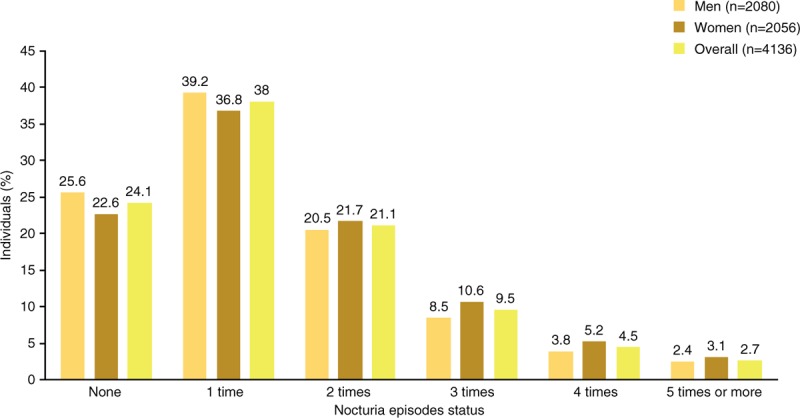
Nocturia episodes in China.

Approximately three-quarters of individuals reported ≥1 episode of nocturia per night (Fig. 4), and similar percentages of men and women were affected (74.4% and 77.4%, respectively). The prevalence of nocturia ≥2 episodes per night was 37.9% (men: 35.2%, women: 40.6%). In both men and women, the prevalence of nocturia (≥1 or ≥2 episodes per night) increased with age. There was 1 exception to the age-related trend: in women aged >60 years, the prevalence of ≥2 episodes per night was lower than that in those aged 55–60 years (54.7% vs 56.7%).

Women with all 3 ICS symptom categories—voiding, storage, and postmicturition—had a lower sexual QOL (SQOL-F mean score 64.5) than those experiencing other ICS symptom combinations (66.8–81.8; Table 4). Sexual QOL was the highest among women without LUTS, with a mean SQOL-F score of 87.2. Among men, the presence of LUTS was associated with reduced IIEF overall satisfaction and, as in women, the greatest negative effect was observed in those with all 3 symptom categories (Table 5). Mean IIEF overall satisfaction scores were 5.5 in men with voiding, storage, and postmicturition symptoms, 6.2 to 7.0 in men with other ICS symptoms, and 7.3 in those without LUTS.

**Table 4:**
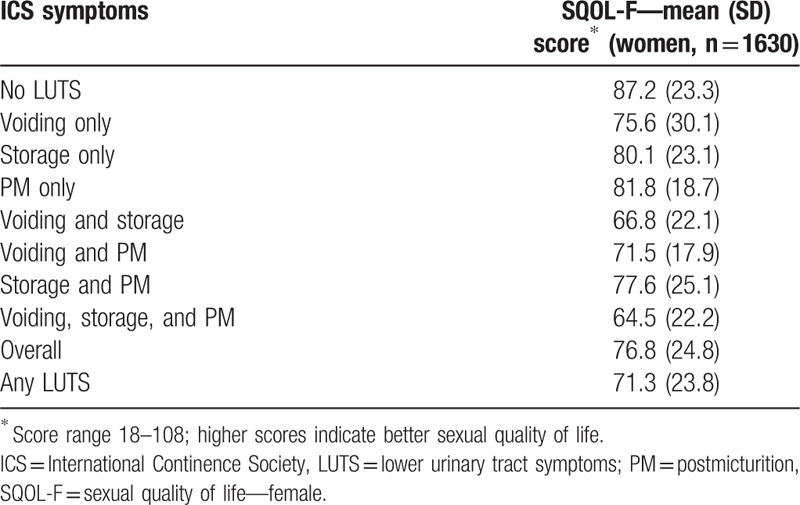
Lower urinary tract symptoms in China according to International Continence Society symptoms and female sexual quality of life.

**Table 5:**
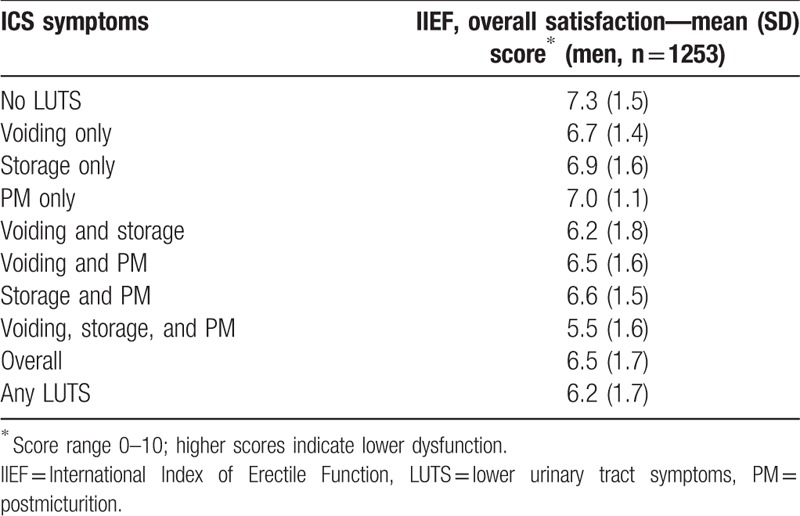
Lower urinary tract symptoms in China according to International Continence Society symptoms and International Index of Erectile Function overall satisfaction.

Lower HRQOL (SF-12) scores were observed in both physical health and mental health domains among individuals with versus without LUTS according to ICS criteria (Table 6). The largest decreases were apparent in those with voiding, storage, and postmicturition symptoms. The presence of LUTS was also associated with higher HADS anxiety and depression scores (Table 7). As with QOL, the greatest impairment was seen among individuals with voiding, storage, and postmicturition symptoms.

**Table 6:**
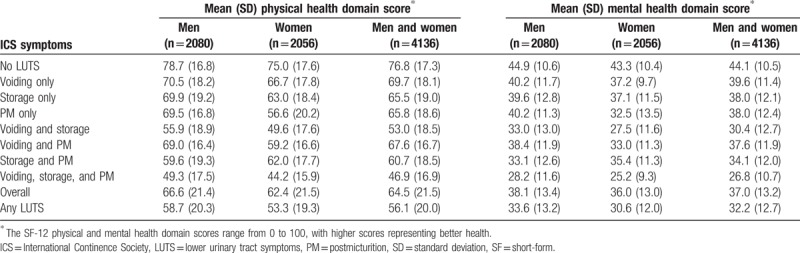
Health-related quality of life score, Short Form-12 (physical health domain and mental health domain) in China by International Continence Society symptoms.

**Table 7:**
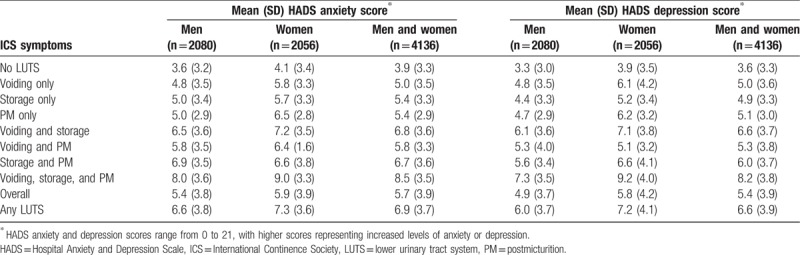
Hospital Anxiety and Depression Scale anxiety score and Hospital Anxiety and Depression Scale depression score in China by International Continence Society symptoms.

**Figure 5:**
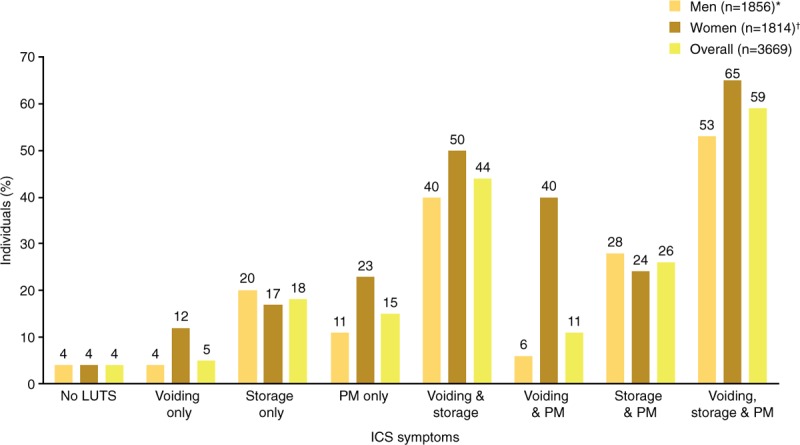
Healthcare according to ICS symptoms: individuals visiting a healthcare professional in China for urinary symptoms. ^∗^∼11% did not answer. ^†^∼12% did not answer. ICS = International Continence Society, LUTS = lower urinary tract symptoms, PM = postmicturition.

The overall percentage of participants with LUTS visiting healthcare professionals for urinary symptoms was 38%, rising to 59% in those with voiding, storage, and postmicturition symptoms (Fig. 5). For individuals with voiding, storage, and postmicturition symptoms, the most common LUTS treatments were prescription medication, limiting intake of fluids, physical therapy, and self-treatment (Table 8). The same treatments were most commonly used by individuals with other ICS symptom combinations. Physical therapy was more commonly used by women than by men.

**Table 8:**
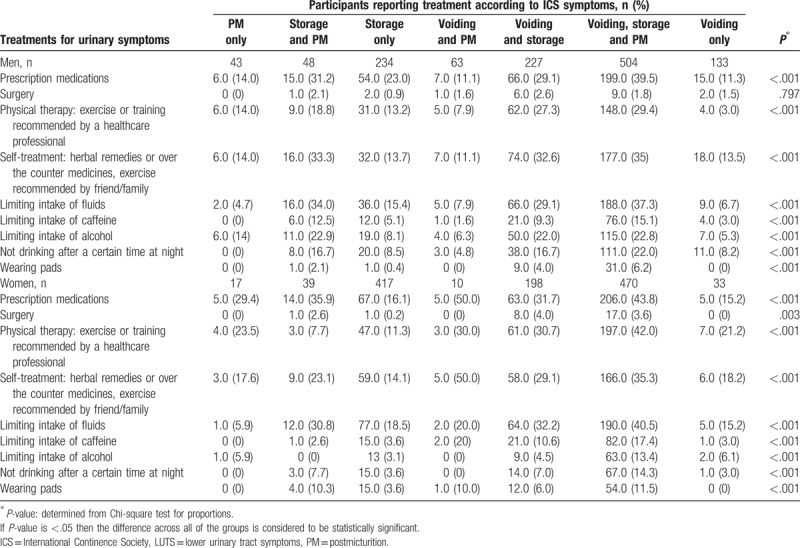
Treatment for lower urinary tract symptoms in China (men and women).

Based on logistic regression results, the presence of comorbid conditions (diabetes and hypertension) and specific urinary symptoms (hesitancy, perceived frequency, nocturia, nocturnal enuresis, urgency with fear of leaking, urge incontinence, and stress incontinence [exercising]) were significant predictors of seeking treatment for urinary symptoms (Table 9).

**Table 9:**
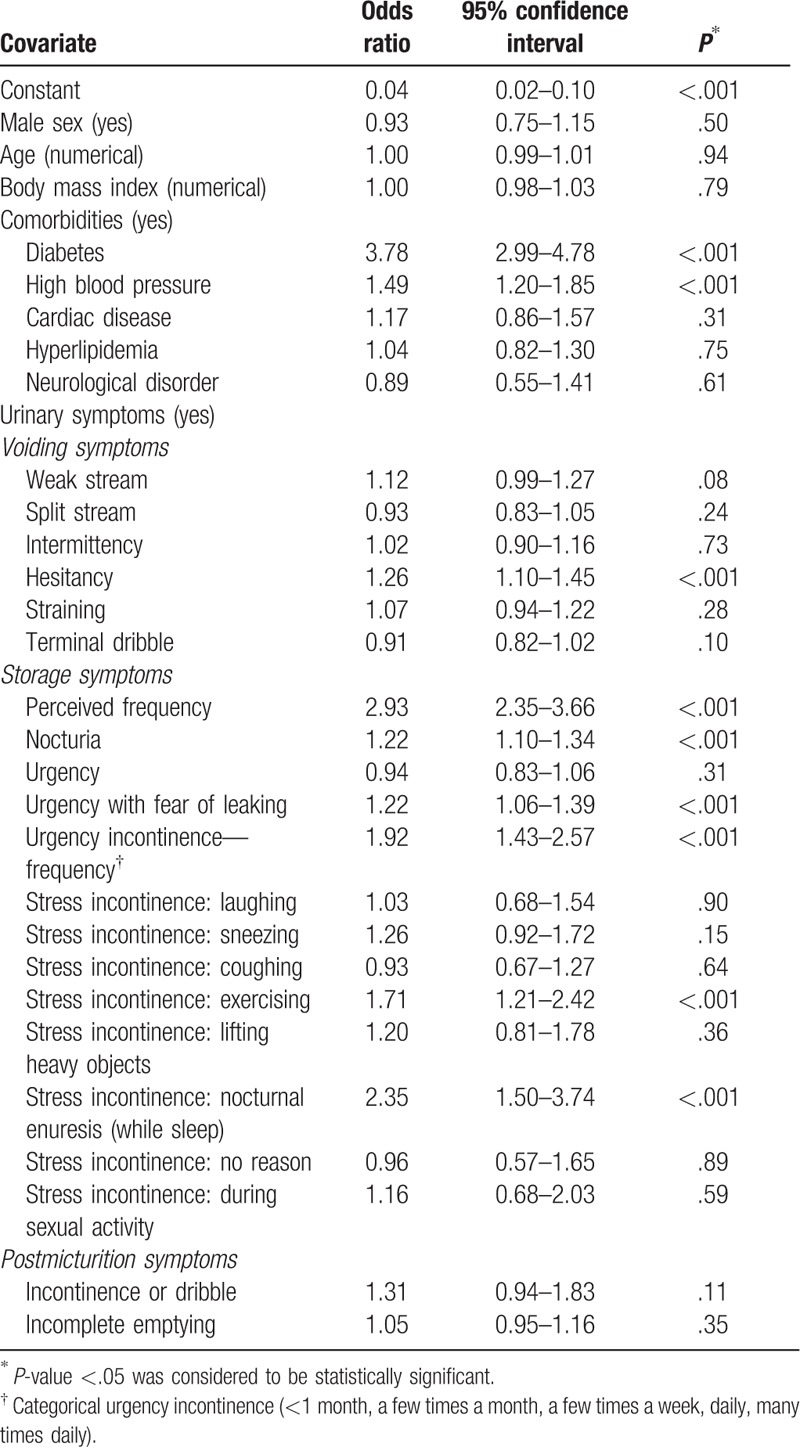
Logistic regression analysis of predictors of treatment-seeking behavior.

## 4 Discussion

This study represents the largest population-based survey to date of LUTS among men and women in China. The results show that LUTS affect more than half of the Chinese population aged ≥40 years, and that the prevalence increases with age. Nocturia was the most common symptom, with approximately three-quarters of individuals reporting ≥1 episode per night and more than one-third reporting ≥2 episodes per night. In a given individual, multiple LUTS are more likely to be present than 1 symptom alone, and bothersome symptoms are common. The overall LUTS prevalence was similar in men and women but, as expected, storage symptoms including SUI were more common in women and voiding symptoms were more common in men. LUTS prevalence was measured by both ICS and IPSS criteria, but the ICS data may be considered as more definitive. IPSS was initially designed to assess men with benign prostatic hyperplasia; it contains only 3 questions on storage and none on postmicturition symptoms; and it is less effective than ICS for distinguishing between individual symptoms.^[3,17]^

A number of previous surveys have assessed LUTS prevalence worldwide and in China, some of which have reported similar findings to the present study. For example, Wang et al^[4]^ reported a prevalence rate of 61% among Chinese adults aged ≥18 years, similar to the prevalence of 64% from the EPIC study that was conducted in 4 European countries and Canada.^[3]^ In both studies, prevalence increased with age and nocturia was the most common symptom. In the Chinese study, there was no difference in prevalence between men and women, whereas in the international study and in contrast to our study, prevalence was slightly higher in women (66.6%) than in men (62.5%). A study in adult Chinese women reported that LUTS affects 56% of individuals, and that the prevalence increases with age.^[18]^ However, some variability in prevalence data has also been observed. A lower LUTS prevalence of 40% was reported in Chinese women aged ≥20 years completing the Bristol female lower urinary tract symptoms questionnaire,^[19]^ while a higher prevalence of LUTS according to IPSS was reported by Yee et al,^[20]^ with moderate-to-severe symptoms occurring in 69% of men aged ≥40 years. Possible contributors to this variability include conducting surveys in different geographical areas, introducing cultural/ethnicity differences; the use of different survey methods; sample size (if too small, results may not reflect the overall population); and demographic characteristics of study populations. Considering the size of the present study, we are confident that our results can be generalized to the overall population in China, although differences between regions cannot be excluded.

The association between age and LUTS prevalence reflects previous observations. The profile of symptoms can change with age—for example, erectile dysfunction and ejaculatory dysfunction are much more likely to be found among men aged over 70 than those aged 50 to 54.^[21]^ The association we found between LUTS and impairment of QOL is also as expected: such associations have been observed in studies performed both in China^[5,8]^ and other countries.^[6,11,22–25]^ It has been reported previously that LUTS may be associated with increased depression/anxiety^[6,11,12,25–27]^ and worsened IIEF score.^[28]^

The presence of LUTS was associated with reduced sexual QOL in women, reduced satisfaction with erectile function in men, higher anxiety and depression scores, and reduced health-related QOL (physical health and mental health domains). All of these effects were greater among individuals with all 3 ICS symptom categories (voiding, storage, and postmicturition) than in those with other symptom combinations. Therefore, LUTS with all 3 ICS symptom categories may be considered to be the most severe form.

OAB is a subset of storage symptoms, so a lower prevalence than that of ICS-defined storage symptoms is in line with expectations. OAB commonly co-exists with other LUTS and it has been shown that men with OAB are likely to report more symptoms and higher severity than those with LUTS but no OAB.^[29]^ An updated OAB definition has been proposed.^[30]^ It would be interesting to see how the use of the new definition (as opposed to OABSS criteria) would affect the prevalence of OAB. In a previous study of OAB in China (conducted in men >50 years of age), the prevalence of 26% was similar to the rate of 24% in the current study.^[31]^ However, a lower prevalence of 2.1% was reported in a community-based study performed using OABSS in Chinese adults aged ≥40 years.^[32]^ Other authors have published relatively low estimates of OAB prevalence: 11% among Chinese individuals aged ≥40 years,^[33]^ and 6% in those aged ≥18 years.^[7]^ In this latter publication, OAB prevalence in men and women aged ≥50 years was 14.8% versus 16.1%, respectively; the authors commented that, had the symptom frequency of urgency not been emphasized, the prevalence of OAB in those aged ≥40 years would have been 19.9%.^[7]^ In the EPIC study, overall OAB prevalence was 12% with similar rates between men and women, although women aged ≥60 years reported more OAB than younger women, while the reverse was true for men.^[3]^ Differences between studies in OAB prevalence could be caused by survey methods. Internet-based methodology brings the risk of participants not understanding questions, while embarrassment can be a barrier to truthful responses during face-to-face interviews. It is possible that ethnic or cultural differences between populations could also contribute to variability between studies.

The associations observed in our study between comorbidities and increased OAB prevalence are broadly in line with expectations, although data from different studies are often conflicting. There are mechanistic, possibly causative relationships between some of the comorbidities and OAB. For example, disruption of the mechanisms that affect the pathophysiology of OAB is seen in neurologic disorders^[34]^ and diabetes can affect bladder function.^[35]^ Smoking can trigger OAB,^[36]^ but while we found smoking to be a significant comorbidity, reports of smoking as a risk factor differ and there may even be differences between the sexes.^[37–39]^ For other comorbidities, underlying risk factors may overlap with the risk factors for OAB (e.g., obesity).

Fewer than half of individuals with LUTS in our study consulted a healthcare professional for urinary symptoms. Embarrassment relating to cultural/social convention has previously been suggested as a reason for reluctance among Chinese women to seek healthcare for urinary incontinence. Additional barriers may include treatment costs, low QOL expectations, and lack of education. Without seeking healthcare, treatment is unlikely to be optimal. Therefore, our data indicate that treatment could be improved and that the impact of LUTS on QOL could be reduced. Education of both the public and physicians could help increase diagnosis and treatment rates. In addition, efforts to reduce stigma could be valuable.^[40]^

Overall, the results for China from our study are consistent with the results for the whole study including South Korea and Taiwan. The clearest difference between China and the overall population is that higher percentages of individuals in China visited healthcare professionals because of urinary tract symptoms. Also, the prevalence of OAB was higher in China than in the other 2 countries.

Strengths of our study include the large sample size, balanced numbers of men and women, and the use of well-established and validated assessments.^[41,42]^ There are also some limitations to consider. The internet-based approach introduces potential for bias because the internet penetration rate was ∼50% in China at the time of the study^[43]^; internet availability may be limited in lower social classes where the unmet needs for LUTS management may be most important. As with all telephone or internet-based surveys, symptom prevalence could potentially be falsely raised by the inclusion of individuals who had a urinary catheter either following urinary tract surgery, which can be associated with temporary LUTS, or for reasons other than urinary tract diseases. Conversely, a lower prevalence of LUTS may be observed if individuals received treatment to control their symptoms. In addition, there is a lack of information to show the extent of coverage across different regions of China and ethnicities. Finally, oestrogen deficiency occurring postmenopause is associated with LUTS in women,^[44]^ but related demographic characteristics such as hysterectomy and menopause status were not collected in our study.

## 5 Conclusion

LUTS affect the majority of adults aged ≥40 years in China, and prevalence increases with increasing age. LUTS are associated with impairment of QOL and mental health, but fewer than half of individuals in China with LUTS seek healthcare for their symptoms. There is therefore a need to improve awareness of the condition, which should improve rates of diagnosis and effective treatment.

## Acknowledgments

The authors would like to thank the participants of the study for their time, and Nanjangud Shankar Narasimhamurthy and Koni Raviprakash for statistical analyses. Editorial support, including writing assistance, was provided by Ken Sutor BSc and Jackie van Bueren BSc of Envision Scientific Solutions, funded by Astellas Pharma Global Development.

## Author contributions

**Conceptualization:** Jian-Ye Wang, Limin Liao, Ming Liu, Budiwan Sumarsono, Min Cong.

**Data curation:** Budiwan Sumarsono.

**Formal analysis:** Budiwan Sumarsono.

**Methodology:** Jian-Ye Wang, Limin Liao, Ming Liu, Budiwan Sumarsono, Min Cong.

**Validation:** Budiwan Sumarsono.

**Writing – review & editing:** Jian-Ye Wang, Limin Liao, Ming Liu, Budiwan Sumarsono, Min Cong.
